# Neck pain: global epidemiology, trends and risk factors

**DOI:** 10.1186/s12891-021-04957-4

**Published:** 2022-01-03

**Authors:** Somaye Kazeminasab, Seyed Aria Nejadghaderi, Parastoo Amiri, Hojjat Pourfathi, Mostafa Araj-Khodaei, Mark J. M. Sullman, Ali-Asghar Kolahi, Saeid Safiri

**Affiliations:** 1 Research Center for Integrative Medicine in Aging, Aging Research Institute, Tabriz University of Medical Sciences, Tabriz, Iran; 2 Research Deputy, Faculty of Medicine, Tabriz University of Medical Sciences, Tabriz, Iran; 3 School of Medicine, Shahid Beheshti University of Medical Sciences, Tehran, Iran; 4 Systematic Review and Meta-analysis Expert Group (SRMEG), Universal Scientific Education and Research Network (USERN), Tehran, Iran; 5 Department of Anesthesiology and Pain Management, Faculty of Medicine, Tabriz University of Medical Sciences, Tabriz, Iran; 6 Department of Persian Medicine, School of Traditional Medicine, Tabriz University of Medical Sciences, Tabriz, Iran; 7 Department of Social Sciences, University of Nicosia, Nicosia, Cyprus; 8 Department of Life and Health Sciences, University of Nicosia, Nicosia, Cyprus; 9 Social Determinants of Health Research Center, Shahid Beheshti University of Medical Sciences, Tehran, Iran; 10 Neurosciences Research Center, Aging Research Institute, Tabriz University of Medical Sciences, Tabriz, Iran; 11 Social Determinants of Health Research Center, Department of Community Medicine, Faculty of Medicine, Tabriz University of Medical Sciences, Tabriz, Iran

**Keywords:** Neck pain, Epidemiology, Risk factor, Narrative review

## Abstract

**Background:**

Neck pain is one of the most common musculoskeletal disorders, having an age-standardised prevalence rate of 27.0 per 1000 population in 2019. This literature review describes the global epidemiology and trends associated with neck pain, before exploring the psychological and biological risk factors associated with the initiation and progression of neck pain.

**Methods:**

The PubMed database and Google Scholar search engine were searched up to May 21, 2021. Studies were included that used human subjects and evaluated the effects of biological or psychological factors on the occurrence or progression of neck pain, or reported its epidemiology.

**Results:**

Psychological risk factors, such as long-term stress, lack of social support, anxiety, and depression are important risk factors for neck pain. In terms of the biological risks, neck pain might occur as a consequence of certain diseases, such as neuromusculoskeletal disorders or autoimmune diseases. There is also evidence that demographic characteristics, such as age and sex, can influence the prevalence and development of neck pain, although further research is needed.

**Conclusions:**

The findings of the present study provide a comprehensive and informative overview that should be useful for the prevention, diagnosis, and management of neck pain.

## Background

Neck pain is a multifactorial disease, and is a major problem in modern society. Although neck pain may not be the most common musculoskeletal disorder, it is still very important [1, 2]. The economic burden of neck pain is remarkable and includes treatment costs, reduced productivity and job-related problems. In 2016, among the 154 conditions, low back and neck pain had the highest health care spending in the United States with an estimated $134.5 billion [3]. In 2012, neck pain was responsible for job absences among 25.5 million Americans, who missed an average of 11.4 days of work [4]. In 2017, the global age-standardised prevalence and incidence rate of neck pain were 3551.1 and 806.6 per 100,000, respectively [5].

There is no one definitive treatment for neck pain. However, different pharmacological and non-pharmacological treatments have been recommended, including laser therapy, massage, acupuncture, yoga, and aquatic therapy [6–8].

The present study aims to provide a narrative review of the most recent data on the epidemiology and trends associated with neck pain. This review will highlight gaps in our knowledge in order to stimulate and focus future research, as well as to help healthcare policy makers and clinicians prevent and control this disease.

## Methods

The PubMed database and the Google Scholar search engine were searched up to May 21, 2021 using the following key words: (“neck pain” OR “neck ache” OR “cervical pain” OR “cervicalgia” OR “cervicodynia”) AND (“epidemiology” OR “global burden of disease” OR “risk factor” OR “biologic factor” OR “psychologic factors” OR “gender” OR “age” OR “genetic” OR “anxiety” OR “depression” OR “stress” OR “neuromusculoskeletal disorder” OR “autoimmune disease” OR “sleep disorder” OR “behavior” OR “social support”). No search filters, for example publication type or date, were used. Our inclusion criteria were studies that were conducted on human subjects and evaluated the effects of biological (e.g. age, gender, and genetic) or psychological (e.g. psychosocial stress, anxiety, and depression) factors on the occurrence or progression of neck pain. In addition, those articles that reported the epidemiological characteristics or burden of neck pain at the global, regional, or national level were also included. In order to consider and address potential biases, two of the authors independently evaluated and reviewed the studies, based on the inclusion criteria, and any discrepancies were resolved by discussion. The data analysis used a qualitative methodology with a narrative synthesis.

## Main text

### Global epidemiology and trends

**Fig. 1:**
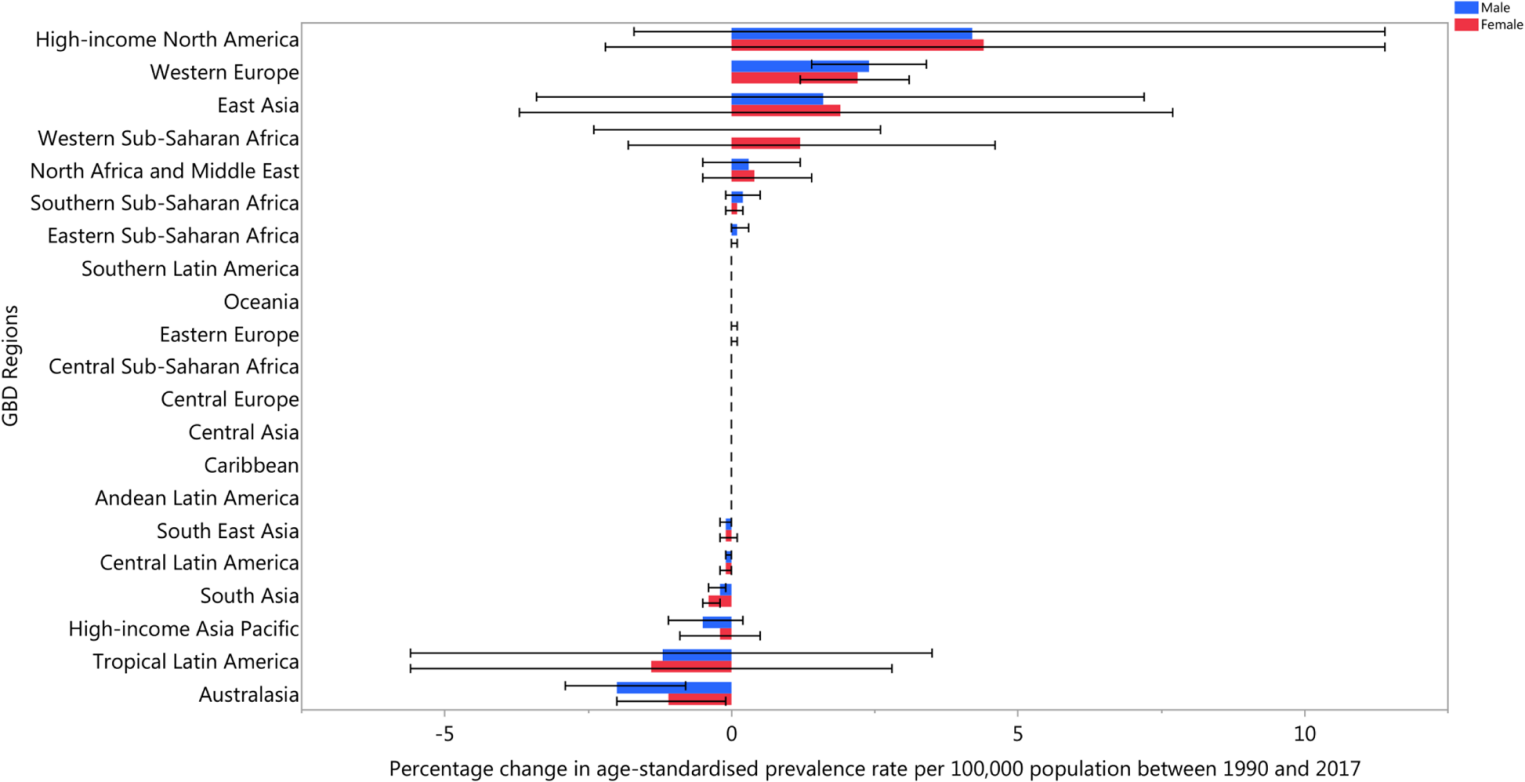
The percentage change in age-standardised point prevalence of neck pain from 1990 to 2017 for 21 Global Burden of Disease regions by sex. (Extracted from the study published by Safiri and colleagues [5])

**Fig. 2:**
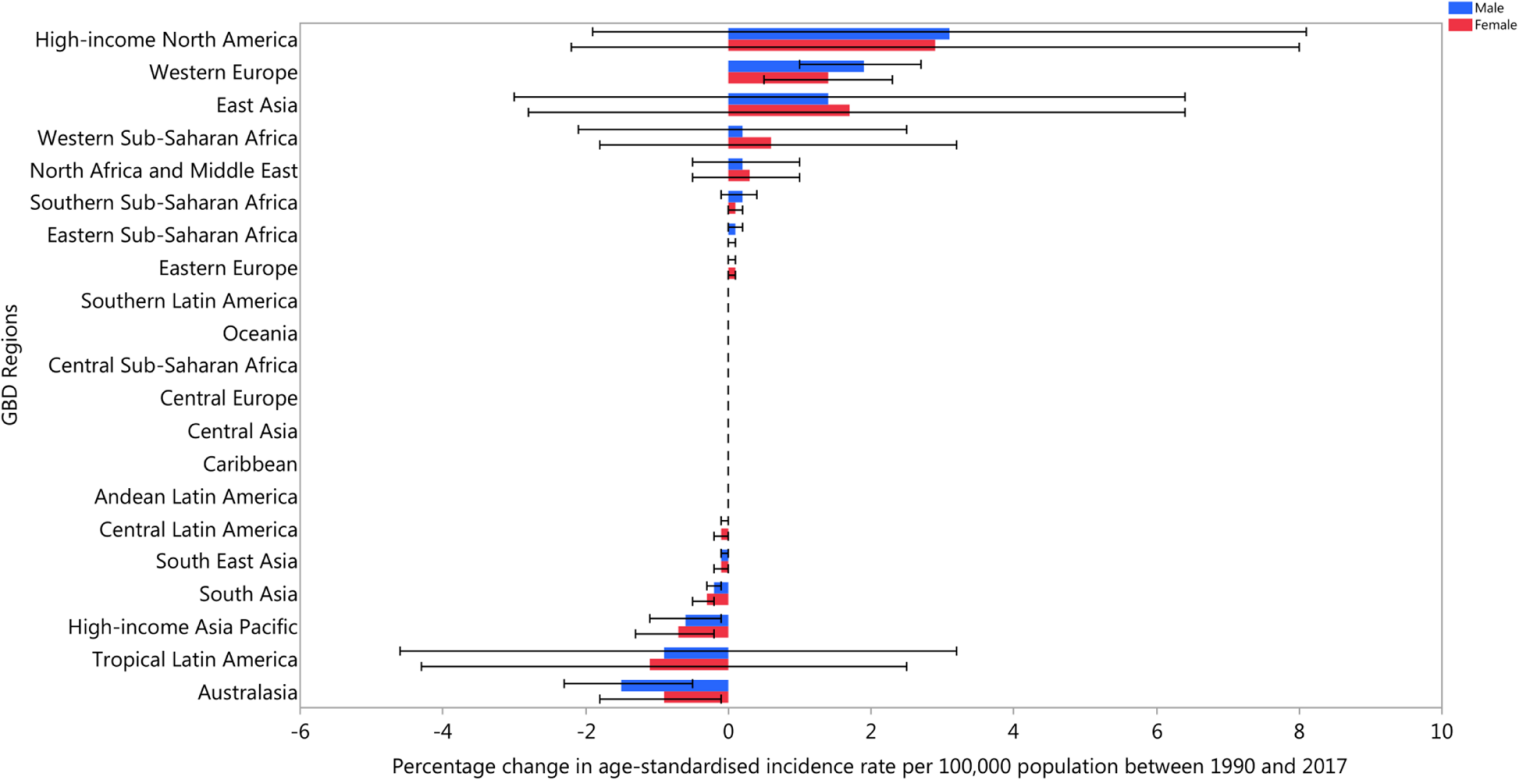
The percentage change in age-standardised annual incidence rates of neck pain from 1990 to 2017 for 21 Global Burden of Disease regions by sex. (Extracted from the study published by Safiri and colleagues [5])

In 2017, the East Asia and Andean Latin America regions had the highest and lowest age-standardised incidence rates, with 1029.0 (910.5 to 1166.1) and 624.0 (550.3 to 708.3) per 100,000 population, respectively. Scandinavian countries, in particular Norway, Finland, and Denmark, had the highest prevalence of neck pain, while Djibouti and South Sudan had the lowest prevalence rates. Over the period 1990–2017, High-income North America had the largest increase in both the age-standardised incidence (3% (− 2 to 7.8)) and prevalence rates (4.1% (− 2 to 11.1)), whereas Australasia had the largest decrease in both the incidence (− 1.1% (− 1.8 to − 0.4)) and prevalence rates (− 1.4% (− 2.1 to − 0.7)) over this period (Figs. 1 and 2) [5].

**Fig. 3:**
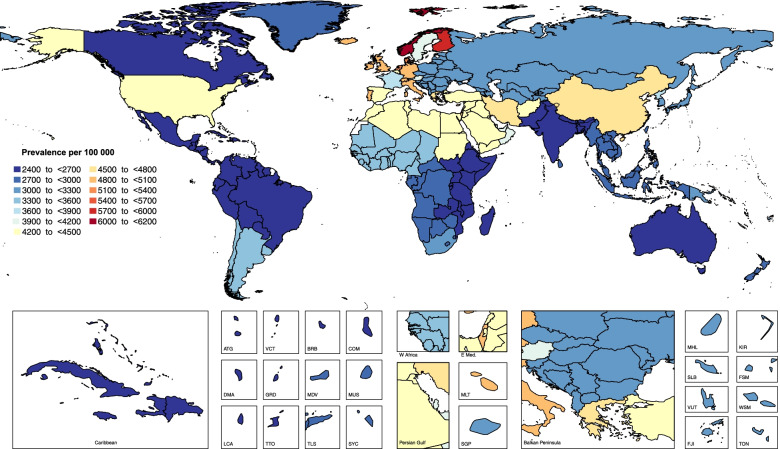
Age standardised point prevalence of neck pain per 100,000 population in 2017, by country. (Extracted from the study published by Safiri and colleagues [5])

In 2017, the national age standardised point prevalence of neck pain ranged from 2443.9 to 6151.2 cases per 100,000 population. As shown in Fig. 3, the countries with the highest age standardised point prevalence estimates (per 100,000) were Norway (6151.2 (5382.3 to 6959.8)) and Finland (5750.3 (5058.4 to 6518.3)), while the lowest estimates were found in Djibouti (2443.9 (2146.4 to 2771.9)) and South Sudan (2449.8 (2149.8 to 2781.1)).

**Fig. 4:**
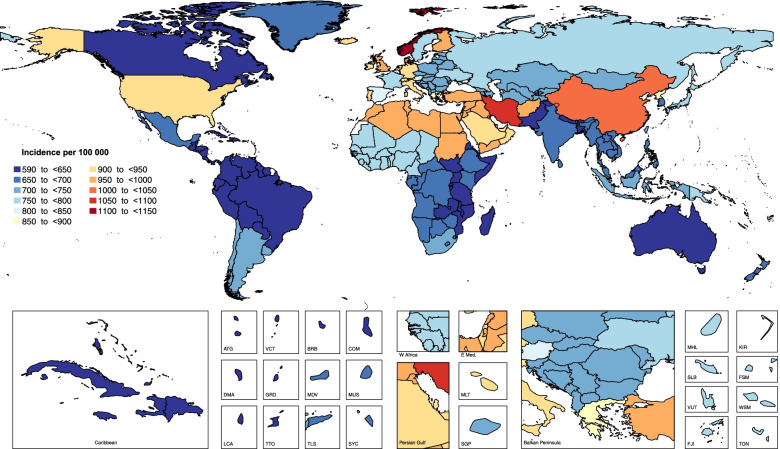
Age standardised annual incidence of neck pain per 100,000 population in 2017, by country. (Extracted from the study published by Safiri and colleagues [5])

Also in 2017, the national age standardised annual incidence of neck pain ranged from 599.6 to 1145 cases per 100,000 population. Figure 4 shows that the highest rates were found in Norway (1145 (1008.8 to 1304.9)) and Iran (1055.5 (927.8 to 1199.6)), while the lowest estimates were found in Canada (599.6 (528.8 to 679.1)) and Bhutan (612.4 (542.1 to 696.3)) (Fig. 4).

**Fig. 5:**
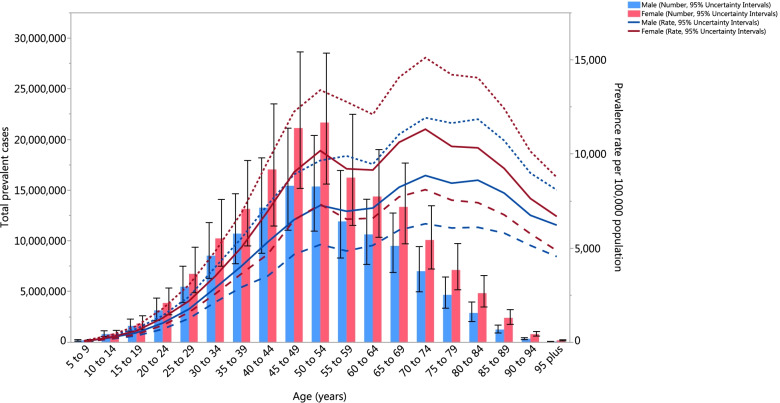
Global number of incident c ases and age-standardised annual incidence rate of neck pain per 100,000 population by age and sex, 2017; Dotted and dashed lines indicate 95% upper and lower uncertainty intervals, respectively. (Extracted from the study published by Safiri and colleagues [5])

The burden of neck pain was higher in females than among males. In 2017, the number of neck pain cases in females was 166.0 million (118.7 to 224.8), while for males it was 122.7 million (87.1 to 167.5) [5]. Furthermore, the number of years lived with disability (YLDs) from neck pain was higher in females (16.4 million (10.0 to 25.1) than among males (12.2 million (7.4 to 18.9) [5]. Globally, in 2017 the age-standardised prevalence of neck pain increased with age up to 70–74, and then decreased with advancing age (Fig. 5). The YLDs pattern across the age groups was relatively similar to that of the estimated point prevalence [5].

### Risk factors

Neck pain is a multifactorial disease. Several population-based studies have explored the role of various modifiable and non-modifiable risk factors for neck pain, such as advanced age, being female, low social support, and a history of neck or lower back pain [9–12]. Since there is a tendency for neck pain to become a chronic problem, it is important to identify risk factors in order to enable prevention and early diagnosis [12]. As neck pain is a multifactorial disease, there are a number of risk factors which can contribute to its development. There is, however, more evidence for some risk factors, such as lack of physical activity, duration of daily computer use, perceived stress and being female [13]. Identifying protective or risk factors, triggers and outcomes can help guide the prevention, diagnosis, treatment, and management of neck pain. The following section describes the research evidence of a number of psychological and biological factors associated with neck pain.

#### Psychological factors

The literature demonstrates a clear link between psychological variables and neck/back pain [14, 15]. A study based on the China Mental Health Survey showed that the prevalence of chronic back or neck pain among people with any type of mental disorders was more than twice that of those without a mental disorder, with a particularly high prevalence among those with mood disorders [16]. Prospective investigations have demonstrated that psychological variables are related to the onset and severity of pain (i.e. acute, sub-acute, and chronic). Stress, distress, anxiety, mood and emotions, cognitive functioning, and pain-related behaviors have all been found to be important factors in the development of neck pain. Although there is not much evidence for personality factors like abuse, they can also be potential risk factors [14, 15].

Overall, factors such as stress, pain catastrophizing, depressive symptoms, low sleep quality, and alcohol consumption might play some role in changing the central pain processing within the spine, brainstem, or cortical levels, which can manifest as remote hyperalgesia [17]. However, further investigations are needed into the role that these cognitive, affective, and lifestyle factors have in central pain processing in non-traumatic neck pain [17]. The four psychological domains (i.e. cognitions, emotions, social and behavioral domains) involved in neck pain were carefully described and explored in depth. Firstly, there is a cognitive component that is comprised of attitudes, beliefs, and cognitions in relation to pain, disability, and perceived health. A second theme is the emotional dimension, in which distress, anxiety, and depression are the most important variables. Thirdly, there is a social dimension, where family and work issues seem to be related to neck and back pain, although the data are less convincing. Finally, a behavioral domain has also emerged, in which coping, pain behaviors, and activity patterns are important elements [14, 18–21].

#### Stress

Stress is related to pain and disability [14, 15]. Perceived stress is a risk factor for neck pain [19, 22]. At least two investigations, with fair methodological quality, have found that adolescents with neck pain had significantly more symptoms of stress than adolescents without neck pain, and that permanent and/or regular feelings of stress was significantly associated with an increased odds of reporting neck pain [23]. Stress may contribute to altered central pain processing at the spinal, brainstem, or cortical levels, which may present as remote hyperalgesia - a condition in which individuals experience an enhanced sensitivity to pain [17, 24]. Moreover, stress acts as a mediator between pain and disability [25, 26].

#### Anxiety

Anxiety is related to different kinds of chronic pain (e.g., neck pain), as well as disability [14, 15, 27, 28]. Neck pain has been found to be comorbid with anxiety [28–30]. Trait and state anxiety were investigated using two different measurement instruments and the researchers found that adolescents with neck pain had higher levels of trait and state anxiety than adolescents without neck pain [23]. Furthermore, anxiety disorders were found to be the second most common comorbid disease associated with neck pain, and specific phobias were the most prevalent problem among those with anxiety disorders [16].

An association between lower pressure pain thresholds (PPTs) and increased levels of anxiety have also been reported. PPTs have been found to be associated with pain intensity, frequency, duration, and disability due to neck pain [31]. People with neck pain have reported higher levels of anxiety [31], and anxiety has also been found to exacerbate pain and disability [25]. However, there are also some discrepancies in the findings, with one study on the components of psychological distress (e.g., stress, anxiety, and depression) reporting that anxiety was not a mediator for pain and disability [26]. Survey-specific estimates have also revealed some inconsistencies in the comorbidity between spinal pain, both back and neck pain, and anxiety disorders. For instance, only 10 out of 17 surveys showed significantly increased odds ratios for chronic neck pain among those with generalized anxiety disorder and agoraphobia/panic disorder. Therefore, there are some variations in the size of this association between studies and between countries, although chronic spinal pain seems to increase the likelihood of comorbid anxiety disorders.

The research has also shown that some specific types of anxiety disorders are more strongly associated with spinal pain than others. Generalized anxiety disorder and post-traumatic stress disorder (PTSD) are more likely to be comorbid with spinal pain than social phobia or panic disorder/agoraphobia [28, 30, 32]. However, research has found neck pain to be more common in patients with mood disorders than among those with specific anxiety disorders [30].

#### Depression

The relationship between depression and neck pain appears to be bidirectional [33]. Mood disorders, especially depression, have been found to be related to chronic neck pain and disabilities [14, 27, 30, 32]. Neck pain is also commonly reported in individuals with underlying depression [13, 29, 34]. Furthermore, a meta-analysis reported that symptoms of depression were associated with high morbidity in neck pain patients [21]. Moreover, a review article suggested that the strongest psychosocial risk factors among respondents with chronic back or neck pain were depressed mood [12] and major depression [30]. A survey study in China showed that mood disorders have a higher comorbidity with neck pain than other mental disorders, and that major depression had the highest comorbidity among all mood disorders [16]. Furthermore, seven studies with fair methodological quality, investigated depression using a total of six different measurement instruments and all studies found that adolescents with neck pain had more depressive symptoms than asymptomatic adolescents [23]. In fact, depressive symptoms may affect central pain processing at the spinal, brainstem or cortical levels, which can be manifested as remote hyperalgesia [17, 35]. Depression and pain might also be risk factors for each other [21]. Several studies have pointed out that psychological stress and the potential obstacles caused by pain may produce immunological changes that eventually results in depression and anxiety [21]. Studies have also found that depression acts as a mediator between pain and disability [25, 26].

#### Cognitive variables

Cognitive factors (i.e. attitudes, cognitive style, and fear-avoidance beliefs) have been linked to increased pain, such as neck pain and disability [14, 15, 27]. Pain cognitions, like catastrophizing and self-perceived poor health, are related to pain and disability [14], as are fear-avoidance beliefs and passive coping [14]. Pain catastrophizing, which is a cognitive factor, may also contribute to altered central pain processing at several levels (i.e., the spinal, brainstem or cortical levels), which may be exhibited as remote hyperalgesia [17, 36]. Research has found that adolescents with neck pain had higher levels of catastrophizing, compared to adolescents without neck pain [23]. In general, people with neck pain report higher levels of catastrophizing than those without neck pain [31].

Another significant cognitive factor is self-efficacy, which has been shown to be related to neck pain [27]. Low pain self-efficacy has been found to be associated with greater functional disability in patients with neck pain [27], although an article by Andias et al. showed that the difference in self-efficacy levels among adolescents with and without neck pain remains controversial [23].

Low resilience is another psychological factor which has been found to be associated with greater functional disability in patients with neck and back pain [27].

#### Sleep problems

The relationship between sleep quality and neck pain is bidirectional, as both can lead to the other [37]. Five investigations, including one longitudinal and four cross-sectional studies, most of which were fair methodological quality, assessed sleep using a total of six different measurement instruments and found some evidence that insufficient quantity and quality of sleep were significantly associated with increased odds of having neck pain [23]. Therefore, sleep management might be a promising intervention for decreasing pain sensitivity and increasing pain modulatory capacity [17, 38].

There is research to suggest that females with low sleep quality are at a higher risk of neck pain onset, but the findings in male have been inconsistent. Furthermore, one high-quality study found no significant relationship between sleep and shoulder pain, indicating that there is either weak evidence or no higher risk [39–41]. Furthermore, poor sleep quality can lead to an increase in the symptoms of depression for people with high intensity neck pain [33].

#### Social support

Loneliness is an important factor in early adulthood and its relationship with neck pain should be further investigated [13]. Neck pain has been found to have a positive relationship with poor general social support [27] and poor social support at work [2]. A negative relationship between neck complaints and actively seeking social support has also been reported [42].

#### Personality

No evidence exists to support the theory of a “pain prone” personality. Furthermore, findings on the relationship between personality traits or disorders and neck pain have been contradictory [14].

#### Behavior

Dangerous behaviors, digital habits, and abnormal eating-related behaviors, such as food insecurity, are common in early adulthood and these behaviors may also contribute to the development of neck pain [13]. Coping styles are the typical manner in which individuals confront and deal with stressful situations, including illness or pain. There are three basic coping styles, which are task-oriented, emotion-oriented, and avoidance-oriented [43]. The relationship between the different coping strategies and pain has been investigated by several studies [44, 45]. Individuals with an avoidance-oriented strategy have a longer duration of neck complaints, since when faced with a problematic situation they seek distraction, avoid thinking about their problem and try to feel better by smoking, drinking, or relaxing [42]. Furthermore, those who react with annoyance or anger tend to have a longer duration of neck complaints [42]. In contrast, there is a negative relationship between seeking social support and neck complaints. In fact, people with neck problems complain of neck pain for a shorter period of time when they share their concerns with others and receive social support and understanding [42].

#### Work-related factors

In a review, the most commonly reported risk factor was working in awkward/sustained postures [12]. Work and study time, workload and body position at work are the most important contributors to neck pain [13]. Work-place characteristics, such as perceived job demands, effort-reward imbalance and coworker support were all significant risk factors [12]. Furthermore, research has found neck pain to be related to low job control, routine work, lack of decision making opportunities, low ability to influence working conditions, low job satisfaction, and high job strain [2]. Furthermore, high job demands and low coworker support are independent risk factors for neck pain and there is also evidence that low decision-making authority is a risk factor for neck pain [46].

There is some evidence that high skill discretion has a protective effect from neck pain [2]. However, working with a computer is considered to be an occupational condition that causes neck pain [19]. Several factors have been suggested to play key roles in the development of computer-related neck pain, including posture, duration working at a computer, psychological stress, repetitive movements, prolonged static loads, and the psychosocial effects of the working environment [19]. Furthermore, there is also an association between neck pain and eyestrain, regardless of other conditions [19].

#### Neuromusculoskeletal disorders

There are a number of diseases and ailments which have been identified as contributing to neck pain. Neuromusculoskeletal disorders affect the bones, muscles, and nerves, and can manifest themselves in several ways. Neck pain is one of the most common and obvious complaints of patients with disorders, such as cervical spondylosis, fibromyalgia, cervical radiculopathy, and whiplash-associated disorders (WADs) [47, 48].

Cervical spondylosis is an umbrella term for a cluster of abnormalities, all of which involve progressive degenerative changes that affect all components of the cervical spine, which is the most frequent neuromusculoskeletal cause of neck pain [49]. In general, neck or occipital pain is known as the first clinical manifestation of degenerative cervical spondylosis. Several factors can lead to cervical spondylosis, such as a congenital narrowing of the cervical spinal canal, cervical osteoarthritis, neck arthritis, trauma, and wearing of the spinal disks. The likelihood of developing cervical spondylosis increases with age, especially after the fourth decade of life, and its progressive nature eventually leads to the involvement of more than one intervertebral disc [50]. The severity of a patient’s signs and symptoms are determinative of the treatment strategy. In addition to pain relievers, non-invasive pain relief techniques (e.g., physical therapy and neck mobilisation/manipulation), and medications can help ease neck pain from cervical spondylosis. Surgical intervention is also effective in preventing the progression of neurologic decline [51].

Fibromyalgia is characterized by chronic widespread musculoskeletal pain and additional symptoms, such as extreme tiredness, sleep disturbance, cognitive dysfunction, and mood problems. Previous studies have shown that genetic and epigenetic factors have a critical role in disease susceptibility. While the signs and symptoms vary greatly, neck pain is the most frequent complaint among patients [52, 53].

Cervical radiculopathy is a type of neck disorder that results from the compression or irritation of the nerve roots in the cervical spine. Nerve root compression can occur due to spondylosis, instability, trauma, or rarely tumors. The clinical manifestations of radiculopathy are broad, but neck and shoulder pain are the primary complications in patients with cervical radiculopathy. The differential diagnosis of cervical radiculopathy, includes cardiac pain, musculoskeletal diseases, infections, and malignancies [54, 55].

Whiplash-associated disorders (WAD) are characterized by a collection of symptoms affecting the neck and are triggered by sudden acceleration-deceleration movements. WADs are characterized by several clinical complications, including neck pain, nonspecific headache, dizziness, and temporomandibular joint pain that are triggered by a sudden force which stretches the neck muscles and tendons. The prevalence of WADs varies around the world, but up to 50% of patients develop chronic pain. Therefore, the prevalence of cervical facet pain is high in whiplash patients [56–58].

#### Autoimmune diseases

Autoimmune diseases are a chronic and clinically heterogeneous group of diseases that occur in immunocompromised individuals. Autoimmune diseases affect various organs and tissues throughout the body. In some autoimmune diseases, the muscles, joints, and nerves can be the target of the immune system, so they are also likely to affect the cervical spine. The most important autoimmune diseases are rheumatoid arthritis, polymyalgia rheumatic, multiple sclerosis (MS), ankylosing spondylitis, systemic lupus erythematosus (SLE), myositis, and psoriatic spondylitis.

Rheumatoid arthritis is a chronic inflammatory disease that primarily affects the bones, peripheral joints, and ligaments, although it can also affect almost every system. Peripheral joint swelling is a common early symptom, and chronic inflammation of the cervical spine is the second most common feature of rheumatoid arthritis, affecting more than half of all patients with rheumatoid arthritis. Neck pain is one of the earliest symptoms to indicate cervical spine involvement in a patient’s rheumatoid arthritis. Nevertheless, occipital headaches and other neurological symptoms may also present in patients with cervical spine involvement [59, 60].

Polymyalgia rheumatica is a relatively common chronic inflammatory disorder that is often associated with giant cell arteritis and is characterized by widespread aches and stiffness of the neck, shoulder, and hip area. The average age of patients with polymyalgia rheumatic is 70 and it is rarely found in anyone under 50 years of age. Therefore, it would appear that age-related immune activation, in response to environmental triggers, may contribute to the development of polymyalgia rheumatica [61, 62].

MS is considered a multifocal inflammatory autoimmune disease that affects the central nervous system (CNS) and often results in the patient experiencing chronic pain. There are a number of different factors, such as age, sex, disease duration, depression, and fatigue, which cause the prevalence of neck pain to differ widely in patients with MS. Neck pain in MS patients can be due to immobility or Lhermitte’s sign, which is defined as a transient short-lasting sensation related to neck movement [63].

Ankylosing spondylitis is a progressive and debilitating form of arthritis that mainly results in inflammation of the joints of the spine. Neck pain is also common in these patients, due to the inflammation of the cervical spine. Recent genetic studies have identified several genes and multiple pathways involved in ankylosing spondylitis. In particular, the activation of specific immune pathways plays a critical role in ankylosing spondylitis pathogenesis [64].

SLE is a severe systemic autoimmune disease that can affect almost any part of the body. Therefore, chronic pain and fatigue are very common in patients with SLE. Inflamed muscles can cause neck and back pain in SLE patients [65].

Myositis is a rare chronic systemic autoimmune disease that is characterized by profound muscle inflammation and progressive muscle weakness. Myositis typically bilaterally affects the skeletal muscles, including the neck, shoulders, hips, and back muscles. Myositis can affect people of any age and, similar to most autoimmune diseases, there is a greater prevalence of myositis in females than among males [66].

Psoriatic arthritis refers to a group of chronic inflammatory joint diseases that develop in some people with psoriasis. Psoriatic spondylitis is a subtype of psoriatic arthritis that affects the spine and causes pain and stiffness in the back and neck [67].

#### Genetic

Genetic susceptibility plays an important role in the development of complex diseases. Unraveling the genetic contributions to complex traits is one of the greatest challenges facing modern medical genetics. The identification of the genetic determinants of complex diseases can help provide some understanding of the pathogenesis of the disease. Twin studies and genome-wide association studies (GWASs) can help to develop insights into the genetic architecture of diseases.

Several population-based twin studies confirm the genetic influence on neck pain. Twin cohort studies compare the concordance rate among monozygotic (MZ) and dizygotic (DZ) twins. The greater similarity of MZ, than DZ, demonstrates the strong genetic contribution to the development of neck pain [68–73]. However, genetic factors seem especially important in the development of neck pain in early adolescence, while their influence could be considered negligible at older ages [68, 72, 74].

There are very few studies which have attempted to unravel the genetic basis of neck pain. GWASs are a powerful approach to identify the genetic loci that predispose individuals to complex diseases. A recent GWAS of the United Kingdom (UK) Biobank participants revealed that there are three genetic loci associated with neck or shoulder pain, including an intergenic region in chromosome 17 for rs12453010, *FOXP2* gene in chromosome 7 for rs34291892 and rs62053992 in *LINC01572* gene in chromosome 16 [75]. However, to the best of our knowledge, only one GWAS study has been published to date and further evidence should be sought to confirm the genetic correlations between these loci and neck pain.

#### Gender

The results of several systematic reviews have demonstrated that gender is a well-studied but ambiguous risk factor for neck pain. Previous studies have considered being female to be a significant risk factor for developing neck pain [11, 76]. Nevertheless, in contrast to previous neck pain articles, recent epidemiological studies have found no meaningful sex differences in the prevalence, incidence, and years lived with disability across the age groups in patients with neck pain [12, 13, 77]. However, the point prevalence of neck pain was higher in females across all age groups [5]. These contradictions are also evident from the articles that report being female to be a weak predictor of developing neck pain, since the age of onset is also important and this may differ between males and females. For this reason, sex-specific meta-analyses are needed to clarify the ambiguous association between sex and neck pain.

#### Age

Aging is the most important risk factor for most chronic pain, so identifying protective and risk factors is critical for raising awareness about effective preventative measures and educational interventions for high-risk groups [11]. The normal anatomy of the cervical spine changes at advanced ages, which can cause neck pain and long-term disability. Neck pain is common among adults, although it can occur at any age. According to the Global Burden of Diseases 2017 study, the point prevalence of neck pain peaked during the middle ages and declined thereafter, with the highest burdens being in the 45–49 and 50–54 age groups for men and women, respectively [5].

## Discussion

In this narrative review, we aimed to provide the most recent data on the epidemiology and trends associated with neck pain, and its attributable risk factors, in order to highlight gaps in our knowledge and to stimulate and focus further research, as well as to help healthcare policy makers and clinicians preventing and controlling this disease. Our narrative review showed that the global epidemiology of neck pain has not changed substantially over the last 30 years. We also found that both biological and psychological risk factors, like age, genetics, past medical history of musculoskeletal disorders, stress, anxiety, and depression can promote the occurrence of neck pain. However, the effects of some other factors, like personality disorders and gender need to be investigated more deeply.

Efforts to identify the potential risk factors for neck pain have been underway for decades. The effects of different types of psychological and biological factors have been presented previously [14], but these often had small sample sizes [9, 76]. Furthermore, there is a paucity of primary research conducted in several areas of the world, which could lead to limitations in the data sources used to estimate the burden of neck pain in different regions [78]. This article placed the risk factors into two main categories, which were psychological and biological factors. However, there are other methods of categorizing the risk factors, such as according to the level of association and preventative potential. For example, risk factors could be divided into those that have a high correlation and are difficult or impossible to prevent, risk factors with a strong correlation that are preventable, and factors that have a low correlation with neck pain. Furthermore, as previously mentioned, several factors (e.g., sleep disturbances and depression) have a bidirectional association with neck pain.

The risk factors for neck pain can be placed into three categories, which are physical, psychosocial, and individual-related risk factors. In a review by Kim et al., they found that most risk factors for neck pain were related to psychosocial, rather than physical characteristics [12]. Neck pain has also been associated with many other comorbidities, such as headaches, dizziness, anxiety, and depression [29]. Nevertheless, there are also some inconsistencies in the research investigating the relationship neck pain has with psychological factors.

There are also several risk factors, like depression and occupational factors that not only have a strong association with neck pain, but are also preventable. Patients with musculoskeletal disorders are the most common disorder that might benefit from rehabilitation services (1.71 billion people (95% UI: 1.68–1.80)) [79]. Furthermore, neck pain is the second most common musculoskeletal disorders with a higher prevalence and incidence among females [5, 79]. Moreover, in 2016, low-back and neck pain were the third largest cause of disability-adjusted life-years (DALYs) in both sexes (87 million (61 to 114)) [80]. Therefore, finding a cost-effective diagnostic method and treatment strategy is important [81]. Furthermore, improvements in the definition and registration of pain conditions, such as neck pain, could make a large contribution to improving health by providing reliable estimates of the burden of neck pain which can lead to appropriate resource allocation and the implementation of preventative and treatment programs [78].

As previously mentioned, work-related factors (e.g., work load, working on a computer, and study time) are risk factors for the development of neck pain. Nevertheless, children and adolescents are also at a risk of developing neck pain. A cross-sectional study of 207 children and adolescents with non-specific neck pain showed that strong flexion of the neck during studying, sitting, watching television, and using smartphones (or other handheld devices) was associated with neck pain [82]. Furthermore, an article by Scarabottolo et al. revealed that physical inactivity in adolescents was associated with an increase in the risk of cervical pain (odds ratio (OR) = 1.49 (1.06–2.10)) [83]. Moreover, using a mobile phone for more than 10 h per week significantly increased the odds of developing neck pain in students aged 10–17 years old (OR = 2.48; 95% CI: 1.29–4.75) [84].

There is now evidence from large epidemiological studies that there is an association between mental disorders and chronic pain. The comorbidity between psychological disorders and pain has implications for the outcome of pain and possibly also for the outcome of the psychological disorders. The relationship between psychological disorders and chronic pain, in the case of co-occurrence, is often complex and cannot be easily interpreted and managed. Although a relationship between chronic pain and psychiatric disorders has been identified, the relationship pain severity has with mood or anxiety disorders is less clear [32]. Furthermore, research in diverse cultures and socioeconomic situations has shown that people with chronic neck pain were more likely to suffer from psychological disorders (e.g., mood, anxiety, and alcohol disorders), compared to those without neck pain [30].

The most prominent limitation of this study is that it had a narrative approach, rather than a systematic review and meta-analysis, so it is difficult to draw precise conclusions. In addition, we only investigated biological and psychological risk factors, although there are other potential risk factors, such as a history of previous neck injury, number of children, past medical history of low back pain, and poor self-assessed health [30, 85]. In addition, we acknowledge that some of the topics covered under biological risk factors may not have been completely balanced. Despite these limitations, this comprehensive review has highlighted important gaps in our knowledge and areas that require further research. Future research should be conducted as systematic reviews, with or without meta-analysis, on specific biological (e.g. gender) and psychological (e.g. type of personality) risk factors, especially those with inconsistent findings, in order to clarify whether or not there is an association.

## Conclusions

**Fig. 6:**
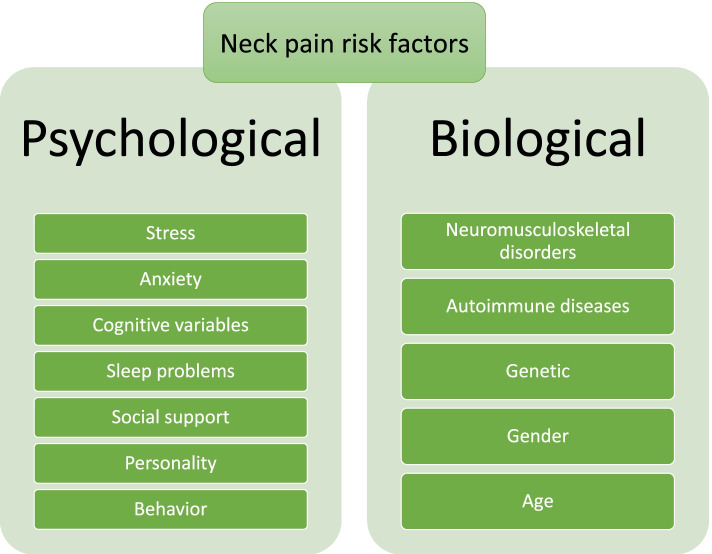
Summary diagram representing the biological and psychological risk factors of neck pain

Neck pain has a high prevalence around the world, although its burden has not changed substantially over the period 1990–2019. Recent literature has shown that psychological factors (e.g., stress, some cognitive factors, and sleep problems) and individual/biological factors (e.g., preexisting neuromuscular or autoimmune disorders, aging, and genetic) both contribute to the development of neck pain (Fig. 6). The relationship between personality types and gender on the risk of neck pain is not yet clear, so further research is needed to investigate the association that neck pain has with gender, personality, and several other psychological factors.

## Data Availability

Not applicable.

## Abbreviations

UI: Uncertainty interval
YLD: Years lived with disability
DALY: Disability-adjusted life years
WAD: Whiplash-associated disorder
MS: Multiple sclerosis
SLE: Systemic lupus erythematosus
CNS: Central nervous system
GWAS: Genome-wide association studies
MZ: Monozygotic
DZ: Dizygotic
UK: United Kingdom
PPT: Pressure pain threshold
PTSD: Post-traumatic stress disorder
OR: Odds ratio
